# Assessment of Fruit and Vegetables Intake with Biomarkers in Children and Adolescents and Their Level of Validation: A Systematic Review

**DOI:** 10.3390/metabo12020126

**Published:** 2022-01-28

**Authors:** Li Yuan, Samuel Muli, Inge Huybrechts, Ute Nöthlings, Wolfgang Ahrens, Augustin Scalbert, Anna Floegel

**Affiliations:** 1 Department of Epidemiological Methods and Etiological Research, Leibniz Institute for Prevention Research and Epidemiology—BIPS, Achterstraße 30, 28359 Bremen, Germany; ahrens@leibniz-bips.de (W.A.); floegel@leibniz-bips.de (A.F.); 2 Unit of Nutritional Epidemiology, Department of Nutrition and Food Sciences, Rheinische Friedrich-Wilhelms-University Bonn, 53115 Bonn, Germany; samuel.muli@uni-bonn.de (S.M.); noethlings@uni-bonn.de (U.N.); 3 International Agency for Research on Cancer (IARC), 69372 Lyon, France; huybrechtsi@iarc.fr (I.H.); scalberta@iarc.fr (A.S.); 4 Section of Dietetics, Faculty of Agriculture and Food Sciences, Hochschule Neubrandenburg—University of Applied Sciences, 17033 Neubrandenburg, Germany

**Keywords:** biomarker, fruit, vegetable, children, adolescents, validation

## Abstract

Fruit and vegetables (FV) are part of a healthy diet and should be frequently consumed already at a young age. However, intake of FV is difficult to assess in children and adolescents due to various misreporting aspects. Thus, measurement of dietary biomarkers may be a promising alternative to assess FV intake more objectively at young age. To date, dietary biomarkers have been primarily studied in adults, and research focused on their usefulness in children is scarce. However, clinical studies have revealed important differences between children and adults, most importantly in their gut microbiome composition, resulting in differences in postprandial metabolism, as well as in food choices and meal compositions that may influence individual biomarker levels. Therefore, the present review aimed to identify biomarkers of FV intake (BFVI) currently available in children and adolescents and to explore whether there are any differences in the BFVI profile above between children and adolescents and adults. In addition, the current level of validation of BFVI in children and adolescents was examined. In total, 28 studies were eligible for this review, and 18 compounds were identified as potential biomarkers for FV intake in children and adolescents. Carotenoid concentration in skin was a valuable biomarker for total FV intake for both children and adult populations. Common BFVI in blood in adults (e.g., carotenoids and vitamin C) showed inconsistent results in children and adolescents. Biomarkers particularly useful in children included urinary hippuric acid as a biomarker of polyphenolic compound intake originating from FV and the combination of *N*-methylnicotinic acid and acetylornithine as a biomarker of bean intake. Further studies are needed to assess their kinetics, dose–response, and other validation aspects. There is limited evidence so far regarding valid BFVI in children and adolescents. Thus, to put BFVI into practice in children and adolescents, further studies, particularly based on metabolomics, are needed to identify and validate BFVI that can be used in future epidemiological studies.

## 1. Introduction

The measurement of fruit and vegetables (FV) intake in children and adolescents is critical for unravelling the association between dietary exposure and current and later adulthood health [1,2]. To date, self-reports and observer-recorded methods are the most common dietary assessment tools. However, self-reported methods, such as 24 h dietary recall and food frequency questionnaires (FFQs), are subject to random or systematic measurement errors due to poor instruments, poor recall, or misreporting of dietary intake [3]. For young children, parents or teachers must assist them to complete a dietary assessment [4]. It is noteworthy that children may consume a significant amount of food outside the home where parents may not be aware [5]. While teachers are serving many children, it may be difficult to record every student’s food consumption accurately [6]. For older children and adolescents, social desirability seems to increasingly bias reporting accuracy [7]. On the other hand, observer-recorded methods such as digital photography or plate weighing offer more valid estimation than self-reported methods [8], but these methods are laborious and are limited to studies with small sample sizes.

Biomarkers of fruit and vegetables intake (BFVI) may aid in the unbiased assessment of FV consumption. To date, numerous interventional and observational studies have been conducted to identify new BFVI in adult subjects and subgroups of men and women [9,10]. In contrast, studies in children and adolescents are rare. Clinical studies have revealed that age, gut microbiota, and diet could result in interindividual variability of postprandial metabolism [11,12,13]. For these reasons, BFVI in young people (2–18 years) and adults may not necessarily be comparable. Margalef et al. [11] discovered a significant reduction in flavanol absorption and phase II flavanol metabolism in adult rats when compared with young rats. This observation may be due to differences in microbiota composition. It is well known that intestinal microbiota undergo maturation from birth to adulthood [12,14]. In addition, many differences at the genus level between young children and adults as well as adolescents and adults have been observed in fecal samples, especially for the genius *Bifidobacterium* [12,13]. Recently, Radjabzadeh et al. [15] observed a shift in branched chain amino acid metabolism, from catabolic pathways in children to biosynthetic pathways in adults. More evidently, dietary behaviors vary between children and parents by nutrients and food groups [16,17], which leads to variable postprandial metabolism as well. For instance, the absorption of carotenoids can be promoted by different dietary fat intakes [18].

Given the above evidence, we suggest that there is a high demand for discovery and validation studies on BFVI specifically in children and adolescents. So far, new potential biomarkers are mainly identified through a single explorative study by metabolic profiling of biofluids or tissues after intake of various foods or based on food chemistry. However, they must be further validated to ensure that they accurately represent the level of intake of the food considered, that the sample type and time of sampling are appropriate for the intended use, and that the analytical method is valid according to current standards. Recently, Dragsted et al. [19] developed a comprehensive biomarker validation scheme based on eight criteria. Therefore, the aims of this extensive literature review are (1) to summarize potential BFVI for children and adolescents, (2) to explore whether there are any differences in the BFVI profile above between adults and children and adolescents, (3) and to examine the current level of validation of candidate BFVI in children and adolescents by following the validation procedure proposed by Dragsted et al. [19].

## 2. Methods

The reviewing process was conducted following guidelines for food biomarkers of food intake as defined by the FoodBAll consortium (BFIRev) [20] and adherence to the PRISMA statement (Preferred Reporting Items for Systematic Reviews and Meta-Analyses) [21]. We registered the review project with OSF Registries (DOI: 10.17605/OSF.IO/9H8UB, https://osf.io/9h8ub/, accessed on 29 October 2021).

### 2.1. Selection of Food Groups

For this review, we took all fruit and vegetable species into consideration, while in the search strategy section, we only listed some of the fruits and vegetables most widely consumed by children and adolescents. Thus, for the fruit group, literature search included fruit, citrus, berry, apple, grape, pear, orange, banana, mango, and pineapple as keywords. The vegetable group consisted of vegetables, tomato, potato, onion, pea, leek, spinach, lettuce, endive, asparagus, kale, brassica, cabbage, and “cruciferous vegetables”.

### 2.2. Literature Search for Biomarkers of Fruit and Vegetables Intake (BFVI) in Children and Adolescents

An extensive literature search was carried out in three databases (PubMed, Scopus, and Web of Science) in January 2021 and updated in August 2021. Keywords included a combination of fruits and vegetables and a group of search terms (fruit* OR vegetable* OR citrus OR berry OR berries OR apple* OR grape* OR pear* OR orange* OR banana* OR mango* OR pineapple* OR tomato* OR potato* OR onion* OR pea* OR leek* OR spinach* OR lettuce* OR endive* OR asparagus* OR kale OR brassica OR cabbage* OR “cruciferous vegetable*”) AND (urine OR urinary OR plasma OR serum* OR excretion* OR blood OR skin OR saliva) AND (biomarker* OR marker* OR metabolite* OR biokinetics OR biotransformation OR bioavailability OR ADME OR metabolom*) AND (child OR youth OR juvenile* OR preschool OR boy* OR girl* OR teenage* OR children OR childhood OR adolescence OR infant* OR infancy OR adolescent*). Keywords were applied in the fields [Title/Abstract] for Scopus and Web of Science, while for PubMed we added an extra [Mesh] term for some specific words, namely, “fruit”, “vegetables”, “Metabolome”, “Metabolomics” “Biomarkers”, “Infant”, “Child”, and “Adolescent”. The search setups for PubMed, Scopus, and Web of Science are presented in Supplementary File S1. The search was limited to papers in English language with no restriction of publication date. Articles including human intervention studies (randomized controlled trials, controlled clinical trials, acute, short-term, or long-term studies) or observational studies (cohort, case-control, cross-sectional studies) were taken into consideration. Search results were imported into EndNote X9 (Thomson Reuters, New York, NY, USA). After removal of duplicates, a primary selection was conducted in Rayyan (Rayyan Systems Inc., Cambridge, MA, USA) based on the relevance of abstract and title. The exclusion criteria were as follows: effect on physiology or clinical endpoint, effect on drug metabolism, food analysis, in vitro studies, animal studies, irrelevant foods, children over 18 years old, fruit and vegetable juice and drink intake, conference abstracts, short communications, editorials, and comments. The screening process was conducted by two reviewers, L.Y. and S.M., who performed the screening independently. A third reviewer, A.F., was responsible for resolving the disagreement. Full texts were obtained for the selected articles and further assessed for eligibility according to their relevance in the identification of BFVI in children and adolescents. Additional papers were identified from a reference list in these papers. The relationships between potential biomarkers and intake of FV were assessed by Pearson or Spearman correlation coefficients. A *p*-value of 0.05 or below was considered statistically significant. The strength of correlation was classified as weak (*r* < 0.2), moderate (0.2 ≤ *r* < 0.6), and strong (*r* ≥ 0.6).

### 2.3. Identification and Characterization of Candidate BFVI

For each potential biomarker identified, a secondary search was needed to retrieve relevant information to assess its plausibility. If a potential BFVI met one or more of the following criteria, it was considered a candidate biomarker [20]: (1) the metabolite has high specificity for the targeted food or food group, (2) the compound is highly characteristic of the food investigated (e.g., abundant in the targeted food compared with other foods), and (3) the marker is not fully specific but could be used in a multimarker approach. The search was conducted with (“the name and synonyms of the compound” OR “the name and synonyms of any parent compound”) AND (biomarker* OR marker* OR metabolite* OR biokinetics OR biotransformation OR bioavailability OR ADME OR metabolom*). In this step, Google Scholar was also used as a search platform, in addition to the above three databases. Additionally, the compounds were searched manually in the online databases HMDB (https://hmdb.ca/, accessed on 15 March 2021), Phenol-Explorer 3.6 (http://phenol-explorer.eu/, accessed on 15 March 2021), and PubChem (https://pubchem.ncbi.nlm.nih.gov/, accessed on 15 March 2021) to determine all the possible dietary or metabolic origins of the candidate BFVI.

### 2.4. Evaluation of the Validation of Candidate BFVI

The secondary search was also used to obtain additional information for assessing the level of validation for biomarkers. A set of validation criteria proposed by Dragsted et al. [19] was applied to evaluate the level of validation of candidate biomarkers. The validation scheme is based on eight questions that encompass biological and chemical aspects: Q1: Is the marker compound plausible as a specific biomarker for the food or food group (chemical/biological plausibility)? Q2: Is there a dose–response relationship at relevant intake levels of the targeted food (quantitative aspect)? Q3: Is the biomarker kinetics described adequately to make a wise choice of sample type, frequency, and time window (time–response)? Q4: Has the marker been shown to be robust after intake of complex meals reflecting dietary habits of the targeted population (robustness)? Q5: Has the marker been shown to compare well with other markers or questionnaire data for the same food/food group (reliability)? Q6: Is the marker chemically and biologically stable during biospecimen collection and storage, making measurements reliable and feasible (stability)? Q7: Are analytical variability (CV%), accuracy, sensitivity, and specificity known to be adequate for at least one reported analytical method (analytical performance)? Q8: Has the analysis been successfully reproduced in another laboratory (reproducibility)?

### 2.5. Risk of Bias and Quality of Study Assessment

The quality of the included publications was assessed by two independent reviewers (L.Y. and S.M.), and differences were resolved after discussion. The adapted Biocross tool was used to evaluate the quality of cross-sectional studies and cohort studies [22]. The tool considered nine dimensions, and each dimension consisted of several items. For every positively evaluated item, 1 point was awarded. Thus, the maximum total score was 25 points. A detailed description can be found in Supplementary File S2. The overall study quality was classified according to the awarded points as follows: poor (≤8), moderate (9–17), and high (≥18). Intervention studies were assessed by the Effective Public Health Practice Project (EPHPP) tool (https://www.ephpp.ca/quality-assessment-tool-for-quantitative-studies/, accessed on 15 March 2021). This instrument is a more generic tool that can be adopted to a wide range of study designs, including randomized controlled trials, controlled clinical trials, and observational studies with and without control groups. The tool assesses 6 domains: selection bias, study design, confounders, blinding, data collection methods, and withdrawals/dropouts. Each domain can be graded as strong (1 point), moderate (2 points), or weak (3 points). For example, if the participants are randomly selected from a comprehensive list or from a source (e.g., clinic) in a systematic manner, or self-referred, the selection bias is scored as 1 or 2 or 3, respectively.

## 3. Results

**Figure 1:**
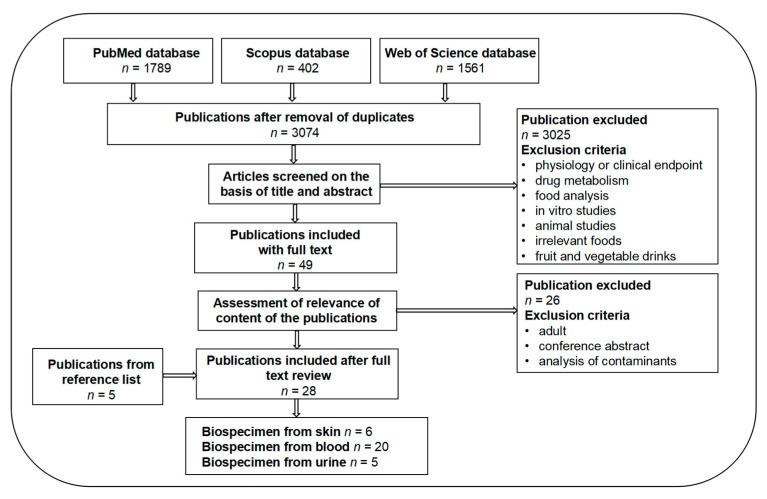
Flowchart of paper selection for BFVI in children and adolescents.

A total number of 3752 papers were retrieved from three databases, while the number of 402 from Scopus was the result after removal of duplicates from PubMed. After screening based on title and abstract, 49 papers were selected for full text reading. A total of 26 additional papers were removed, including 2 conference abstracts, 8 manuscripts regarding adult subjects, and 16 investigating pesticide contaminants. Through full text reading, 5 more papers were identified from the reference lists of these included articles. Subsequently, a total of 28 papers qualified for inclusion in the present review. Six of these papers investigated potential BFVI from skin samples, 20 from blood and 5 from urine, including 1 with both blood and urine samples and another 2 with both blood and skin samples. A flowchart of the selection of the articles is presented in Figure 1.

A summary of the retained literature and the list of the potential biomarkers in children and adolescents is presented in Table 1, including information related to the characteristics of the subjects and study design. As indicated in Table 1, 20 cross-sectional studies, 1 cohort study, and 7 intervention studies reported associations between FV intake and plasma, serum, urine, and skin biomarker concentrations in children and adolescents. All studies except 1 for Lau et al. [23] aimed at verifying or assisting FV intake assessment instruments by using potential biomarkers on the basis of hypothesis-driven targeted metabolomic approaches. Lau et al. [23] used an untargeted high-throughput nuclear magnetic resonance spectroscopy to identify novel biomarkers. There were barely any data in the literature on potential BFVI for specific fruits or vegetables consumed at a young age. Only a few intervention studies investigated associations between wild blueberry, amaranth, cowpea, and *Momordica cochinchinensis* consumption and metabolites in children and adolescents, with 1 exception (papaya) conducted in a cross-sectional study. In total, 18 different potential BFVI were investigated in children and adolescents. The number of potential BFVI measured per study varied from 1 to 6.

### 3.1. Skin Biomarkers

Six of the included studies involved carotenoid measurements in skin to evaluate or verify FV intake in children and adolescents. The ages of the included children ranged from 2 to 17 years. The skin carotenoid concentrations were measured by two noninvasive, quick, and optical technologies, resonance Raman spectroscopy (RRS) (*n* = 5) and pressure-mediated reflection spectroscopy (RS) (*n* = 1). Correspondingly, they were compared with FV consumptions, which were assessed by various tools, for example, 24 h recalls, fruit and vegetable intake frequency questionnaire, food frequency screener, and liking survey. Skin carotenoids measured by RRS were positively associated (*r* > 0.35, *p* < 0.05) with total FV consumption [24,25,26,27,28], except in a study in which FV intake was assessed by the National Cancer Institute’s All-Day Fruit and Vegetable (NCI FV) Screener [26]. In this study, the authors used two tools to assess FV intake—NCI FV and 24 h dietary recall (24H FV)—and they suggested that the NCI FV screener is not a valid tool for FV intake assessment [26]. RRS scores were also verified by serum and plasma carotenoid concentrations separately in two studies [24,27], in which blood carotenoids were measured by high-performance liquid chromatography. Skin carotenoids measured by RRS were strongly correlated (*r* = 0.62, *p* < 0.001) with both serum and plasma carotenoids. While Martinelli et al. [29] applied Veggie Meter (VM), a widely used RS device, to assess skin carotenoids, they only found weak correlations (*r* = 0.174, *p* = 0.042) between total vegetable consumption and VM scores. For the fruit group, no significant correlations were observed.

### 3.2. Blood Biomarkers

In blood samples, carotenoids and their derivatives and conjugates were the most commonly reported metabolites (19 out of 22; 86%) after consumption of individual or total fruit and vegetables, including α-carotene, β-carotene, lycopene, lutein, zeaxanthin, and cryptoxanthin. In some studies, zeaxanthin was included in the content of lutein, because of limitations in their chromatographic separation. The second most frequently identified metabolites were vitamins and their derivatives, including vitamin A (retinol), vitamin C, α-tocopherol (the most biologically active form of vitamin E), and 5-methyltetrahydrofolate (vitamin B family members). In addition, acetylornithine, which belongs to the class of N-acyl-L-alpha-amino acids, was identified in serum samples after FV consumption [23].

However, the usage of blood carotenoids to assess FV intake is not always consistent in children and adolescents from the included papers. Four studies [41,42] demonstrated that carotenoid concentrations in plasma or serum were significantly correlated with total FV intake. Notario-Barandiaran et al. [32] showed that serum carotenoids correlated stronger with vegetable intake (*r* = 0.33) than fruit intake (*r* = 0.19) in male adolescents in Spain [32]. Moreover, Nguyen et al. [27] showed that total plasma carotenoids were significantly correlated (*r* = 0.49, *p* = 0.003) with fruit consumption but not with vegetable intake. Similarly, Hillesheim et al. [35] showed that plasma β-carotene was significantly related (*r* = 0.36, *p* < 0.05) to total vegetable intake and dark green and orange vegetables. Comparable research was conducted in serum, and the authors demonstrated that green-yellow vegetables and total FV intake were significantly correlated (*r* = 0.205, *p* < 0.047) with β-carotene [36,37]. On the other hand, Biltoft-Jensen et al. [30] observed that vegetable intake and α-carotene showed the strongest correlation (*r* = 0.43, *p* < 0.01), and β-carotene was more related to juice intake (*r* = 0.32, *p* < 0.01) [30]. Meanwhile, Prasad et al. suggested that serum α-carotene and β-carotene levels were predominately determined by root vegetable serving [38]. Additional studies suggested that plasma β-cryptoxanthin was most strongly correlated with fruit intake [30,36]. For individual FV, papaya intake is the best predictor (*r* = 0.41) of plasma β-cryptoxanthin concentration [39]. Intake of gac fruit (*Momordica cochinchinensis*) significantly increased the plasma concentration of α- and β-carotene, lycopene, and zeaxanthin [40]. Cowpea leaves, a kind of green leafy vegetable, significantly increased serum β-carotene [41,42].

Four papers involving the usage of vitamin C observed a correlation with overall FV intake, except Vandevijvere et al., who found an inconsistent relationship between vitamin C levels in blood and vegetable intake in adolescents [43]. However, among the four studies, only two took vitamin C supplementation into consideration [44,45]. Vitamin A was determined to connect with gac fruit [40], amaranth [41], cowpea leaf [42], and orange fruit intake [46], respectively. Collese et al. [45] suggested to use the combined plasma vitamins (combined in a score of plasma vitamin recommendations) including β-carotene (provitamin A), retinol, α-tocopherol, and vitamin C to assist total FV intake evaluation in 6- to 10-year-old children [45].

### 3.3. Urinary Biomarkers

Five studies investigated urinary biomarkers of FV intake in children and adolescents [23,47,48,49,50]. In the sole untargeted metabolomic study in this list, five compounds were reported to be associated with FV consumption [23]. They found that hippuric acid was positively associated with both fruit and vegetable consumption, and proline betaine, *N*-methylnicotinic acid (NMNA), and *scyllo*-inositol were positively associated with fruit intake, while acetate was positively associated with potato intake. The urinary hippuric acid excretion and FV intake relationship was confirmed in another study conducted by Krupp et al. [49]. Confounders, including coffee, tea, cocoa, and whole grain intakes, were adjusted in the regression models, and it turned out that the hippuric acid and FV association was even stronger after adjustment in adolescents. Penczynski et al. [50] showed that flavonoid intake from FV correlated with hippuric acid excretion in adolescents, but it is noteworthy that in sensitivity analyses urinary hippuric acid excretion was similarly associated with FV intake and flavonoid intake from FV. In addition, they found that covariates such as coffee, tea, and cocoa intake did not change the correlation between hippuric acid excretion and flavonoid intake from FV. They suggested that urinary hippuric acid is a promising biomarker for flavonoid intake from FV in adolescents. In another study, Barfoot et al. [48] showed that consuming wild blueberry alone could also increase urinary hippuric acid and dihydro caffeic acid 3-O-sulfate. Jones et al. [47] found that overnight urinary potassium correlated significantly with FV intake (*r* = 0.12, *p* = 0.03) as well.

### 3.4. Evaluation of Level of Validation of Candidate Biomarkers

The criteria of plausibility described previously [20] were applied to potential BFVI, and the reasons for including or excluding them as candidate biomarkers are presented in Table 2. Carotenoids and vitamin C were associated with total FV intake instead of specific FV intake, so we discussed them separately. Table 3 summarizes the results of the validation level of candidate biomarkers for specific FV intake based on the eight questions proposed by Dragsted et al. [19].

### 3.5. Risk of Bias and Quality of Study Assessment

The quality assessment of the publications is summarized in Supplementary File S3. The quality scores of 19 cross-sectional and cohort studies ranged from 13 to 23. Their average quality was moderate (mean = 17.4 ± 2.7). The quality of 9 studies was scored as high, and 10 were scored as moderate. The most common reasons for a low-quality ranking were that laboratory staff was not blinded for the analyses, and that reproducibility of the laboratory analysis was not assessed. As for the 7 intervention studies, 2 of them were rated to be of high quality, 4 moderate, and 1 poor. The reasons for the low-quality rating were that they failed to select representative individuals of the target population and there was missing information regarding control of confounders.

## 4. Discussion

### 4.1. Total FV Intake Biomarkers

The results of our systematic review support the use of the skin carotenoid measured by the RRS method to assess FV intake in children and adolescents. Covariates such as age, ethnicity, BMI, weight, waist circumference percentiles, energy intake, and fat intake did not seem to confound the observed associations significantly. The preferred body site for measuring skin carotenoids is the palm of the hands because the skin has lower melanin levels and is less affected by other factors, such as ethnicity or sunlight exposure. Ethnic affiliations were diverse in RRS measurement studies with two of them including more than 50% nonwhite children, and two others more than 20% nonwhite children. In summary, skin carotenoids detected by RRS technology is a valid biomarker to assess dietary FV intake not only in adults but also in children and adolescents [64].

In comparison, the use of serum or plasma carotenoid concentrations to assess total FV intake should be carefully considered in children and adolescents. Associations between biomarkers and reported dietary intake varied widely between studies, which confirms a previous systematic review of studies conducted in adults by Baldrick et al. [9]. They included 63 intervention studies that investigated plasma or serum biomarkers for total FV intake, and all but 6 detected a significant increase in plasma or serum carotenoids after intervention. Possible reasons for a moderate blood carotenoid response to FV intake could be the fact that carotenoids have a long elimination half-life after absorption, and short-term kinetics in blood may be buffered by carotenoids stored in body tissues [65]. In addition, provitamin A carotenoids (β-carotene, α-carotene, and cryptoxanthin) undergo extensive conversion to retinyl esters in the mucosa, and dietary fat plays an important role in biotransformation [66]. Tissues such as liver, adipose tissue, skin, and eye in humans accumulate carotenoids as well [67]. Therefore, the blood concentration of carotenoids may not be the optimal measure to quantify FV intake.

In general, there are three hypothetical factors that influence the measured carotenoid concentrations of the skin: (1) translocation from subcutaneous or visceral fat tissue, blood, and lymph [68]; (2) secretion onto the skin surface via sweat glands and/or sebaceous glands [69]; and (3) topical application of antioxidants in the form of creams and lotions [70]. Blood and skin levels of the carotenoids increase during supplementation; however, increases in skin are delayed. After cessation of supplementation, carotenoid levels in blood decrease faster than in skin [71]. It is suggested that LDL receptors in skin may play a role in the carotenoid uptake [71], and an overall gradient is present within skin layers [68]. That may be the possible reasons that carotenoid concentration in skin is less variable than that in blood, although the usual intake of carotenoid may alter its concentration. However, insight mechanism still needs to be investigated. As a result, skin carotenoids are thought to be a more stable indicator of long-term FV intake, compared with plasma or serum carotenoids.

In a systematic review and meta-analysis of an RCT published up to December 2013, vitamin C is one of the preferred indicators of group FV intake in adults [72]. However, our systematic review provides inconsistent evidence for the usage of vitamin C in children, because Vandevijvere et al. [43] observed that plasma vitamin C had a very weak relationship with vegetable intake in adolescents. Therefore, it remains prudent to use plasma vitamin C as a surrogate marker of overall FV intake in adolescents.

### 4.2. Candidate Biomarkers for Intake of Specific Fruit and Vegetable

Metabolites such as individual carotenoids, α-tocopherol, 5-MTHF in blood and potassium, and acetate in urine are ubiquitously present in plant origin foods or nature [53,54,73], thus they are not suitable as biomarkers for a specific type of FV. Although papaya is indeed one of the richest sources of β-cryptoxanthin, in a study with 159 Costa Rican adolescents [39], this compound was also found in other orange- or red-colored foods, such as orange, mango, apricot, sweet peppers, and pumpkin [18,74]. Vázquez-Manjarrez et al. mentioned that the blood concentration of lycopene is more likely to reflect the intake of tomato products [75]. However, it is noteworthy that lycopene is permitted as a food colorant by the European Union (EU) since 2008 [76]. The main food applications of lycopene include soft drinks, flavored milk products, bakery products, and blancmanges [77]. According to the European Food Safety Authority (EFSA), the average exposure of lycopene from both food colorant and natural occurrence was higher in children than in adults and only slightly lower than acceptable daily intakes (ADIs) (0.5 mg/kg body weight) [78]. Therefore, using lycopene concentration may overestimate tomato intake.

Other compound concentrations also increase after FV intake in children and adolescents, namely, hippuric acid, dihydro caffeic acid 3-*O*-sulfate, proline betaine, NMNA, scyllo-inositol in urine, and acetylornithine in serum. They are kept as candidate BFVI, and we will discuss them in the following text separately.

Krupp et al. [49] reported that urinary hippuric acid is a promising biomarker for FV intake in children and adolescents. Penczynski et al. [50] successfully applied this methodology to assess flavonoid intake from FV in a DONALD cohort study including 287 healthy adolescents. They suggest that hippuric acid can be a reliable indicator of flavonoid intake from FV in adolescents [50]. Though hippuric acid excretion is significantly associated with both FV and flavonoid intake from FV, it may be a more promising biomarker for polyphenolic compound intake from FV in children and adolescents, given the fact that hippuric acid is predominantly synthesized from both various flavonoids (for example, catechin, quercetin, kaempferol, and myricetin) and nonflavonoid polyphenolic compounds (for example, chlorogenic acids, caffeic acids) [59]. It is well known that the majority of total polyphenols come from nonfruit and nonvegetable sources, such as coffee, tea, and cocoa. For this reason, hippuric acid was considered a candidate biomarker for polyphenol-rich food intake in adults [79] However, children and adolescents have lower consumption of coffee, cocoa, and tea than adults, and thus the excretion of urinary hippuric acid in children may indeed mainly depend on their FV consumption. On the other hand, controlling the consumption of tea, coffee, and cocoa is recommended in adolescents with considerable intake levels when using hippuric acid to assess polyphenolic compound intake from FV. To date, kinetic and dose dependence information regarding hippuric acid excretion after polyphenolic compound intake from FV in children and adolescents is lacking. Regarding analytical aspects, hippuric acid is stable at room temperature (25 °C) for 2 weeks and at 0 °C for 1 month [80]. Both Penczynski et al. and Krupp et al. used the method provided by Tomokuni et al. [81] with slight modification. Hippuric acid measurements were performed in triplicate, and the arithmetic means of the three measurements were used in the analysis. Additional studies on dose–response and time–response are needed to reach the full validation according to all criteria. The results of validation level assessment are presented in Table 3. Dihydro caffeic acid 3-*O*-sulfate shares the same precursors with hippuric acid, such as quinic acid, but goes through different metabolism processes [59]. In this case, dihydro caffeic acid 3-*O*-sulfate may be considered in combination with hippuric acid to assess the polyphenolic compounds. However, the robustness and other aspects of validation of the combined biomarker need to be investigated in the future.

Proline betaine has been identified as a potential biomarker of citrus fruit intake in many independent studies on adult subjects [60,82]. Though proline betaine has been detected in a variety of foods, such as seafood, vegetables, meat, and coffee, its concentration in these foods is quite low, except in Chinese artichoke, which contains a similar amount of proline betaine as citrus juice [83]. High excretion of proline betaine in urine can thus be due to citrus juice and Chinese artichoke consumption. Orange is the most abundant source of proline betaine among the citrus fruit. Since Chinese artichoke is a rather rare vegetable and not part of the daily diet, proline betaine may specifically predict citrus fruit consumption. The time–response [60], dose–response [84], and robustness [60] of proline betaine being a biomarker of citrus fruit intake have been well studied in adults. In the only study involving children, citrus fruit were not distinguished with the general fruit group in the food frequency questionnaire, and proline betaine was found to be correlated with fruit intake [23]. Based on the above evidence, proline betaine may also be a plausible biomarker for citrus fruit intake in children, but its robustness and other aspects of validation need further investigation.

Lau et al. [23] reported that both urinary NMNA and serum acetylornithine were correlated with fruit intake, and additionally, they were associated with each other (*r* = 0.23). On the other hand, NMNA, also known as trigonelline, is found in high amounts in coffee, fenugreek, and common pea. The only fruit containing trace amounts of NMNA is muskmelon [85,86]. Posma and colleagues [61] conducted an intervention study to identify novel dietary metabolites related to apples, peas, and onions with a combined spectroscopy strategy in adults. Urinary NMNA concentration showed a dose-dependent increase after the consumption of peas. Another randomized controlled intervention study conducted by Perera et al. found serum acetylornithine and NMNA to be markers of dry beans intake based on a test of 46 middle-aged men [87]. Based on the above evidence, the relationship between NMNA and fruit may result from the poor quality of the food frequency questionnaire. Lau et al. [23] only classified foods into 11 general items, and peas and beans were not taken into consideration. The important confounder for the relationship between NMNA and acetylornithine and intake of peas and beans is coffee intake. Nevertheless, in children with restricted coffee consumption, the combination of NMNA and acetylornithine may still be a plausible biomarker to assess general bean intake. However, further studies regarding its robustness, time–response, and dose–response are needed.

*Scyllo*-inositol is one of the nine possible stereoisomers of inositol, rare in nature, but present in some plants, such as barley seeds [88] and chrysanthemum buds [89]. It has been determined that coconut milk is the most abundant source containing 0.05 mg/L *scyllo*-inositol on average [90]. In this case, *scyllo*-inositol may be a putative biomarker for coconut fruit intake. However, it is still not clear whether *scyllo*-inositol is solely related to coconut intake. On the other hand, *myo*-inositol, another inositol stereoisomer widely spread in nature, could increase *scyllo*-inositol excretion in the urine by humans following oral administration of *myo*-inositol (for example, cereal and sweetener). Consequently, it might suggest that *myo*-inositol is converted into *scyllo*-inositol [63]. The robustness of urinary *scyllo*-inositol is thus not well established. *Scyllo*-Inositol was measured by a well-established method, ^1^H NMR spectroscopy. On the other hand, no data are available regarding the other aspects of validation in children and adolescents.

## 5. Strengths and Limitations

As far as we know, this is the first extensive literature review to study biomarkers of FV intake available in children and adolescents to date. This systematic review was conducted under the well-defined criteria of the BFIRev protocol. The current study can provide researchers an overview of the current level of validation of candidate BFVI in children and adolescents. However, there are still some limitations. (1) Some of the included studies were performed with low quality, but the overall quality was still moderate. (2) It is difficult to compare some biomarkers between young people and adults, since data for young people are scarce. (3) This review was constrained to biomarkers from skin, urine, and blood samples, and there may be other promising specimens, such as breath, red blood cells, and hair containing biomarkers, but research regarding these biospecimens is not common.

## 6. The Way Forward

Based on the included studies in this literature review, only a very small number of predefined metabolites (e.g., carotenoids, vitamin C) were identified through hypothesis-driven approaches in most of the articles. The value of food metabolome for dietary exposure assessment still needs to be investigated extensively. An untargeted metabolomic profiling strategy on a high-resolution full-scan MS can cover a wide range of metabolites in a sample. However, this approach has limited reproducibility, and the data processing is complex and must be coupled with advanced multivariate statistics. Indeed, due to a lack of standards, identification of metabolites may be challenging. While targeted analyses have great reliability, selectivity, and sensitivity and produce quantitative data, they only assess a limited number of known metabolites, which may increase the risk of overlooking the metabolomic response of interest. Therefore, efforts have been made to extend the coverage and throughput of targeted metabolite quantification to overcome these limitations. To data, new strategies, such as SWATHtoMRM, which utilizes the broad coverage of SWATHMS technology to develop a high-coverage targeted metabolomic method, have been developed [91]. After evaluating, it has great reproducibility and high accuracy. Therefore, the application of large-scale targeted metabolomics may be a promising approach for novel biomarker discovery in the future. However, the application of biomarkers on dietary intake assessment is still challenging due to their high variation, low producibility, reliability, and robustness. First of all, metabolite identification, coverage, and reproducibility are influenced by sample processing, storage, instrument type, and the data analysis methods used. To date, no standardized and consensus protocols and pipelines exist to develop and validate dietary biomarkers. Moreover, biomarker identification and validation are limited by the lack of sufficient authentic chemical standards and referential spectra. Second, interindividual variation due to differences in host genetics, gut microbiome, and physiologic status, as well as interindividual variation because of half-life of metabolites, exists, which makes dietary biomarkers less reliable. Furthermore, the consumed food is metabolically transformed by endogenous processes or the gut microbiota. This leads to chemical byproducts that are very different from the ones originally ingested in the food. Therefore, to find better food-specific biomarkers, it is important to know more about the pathways through which these chemical constituents are biologically transformed. It has been previously suggested that metabolomics should move beyond biomarkers and towards mechanisms [92]. Ultimately, dietary biomarker discovery can provide hundreds of candidate biomarkers, but these biomarkers need to be thoroughly validated to be meaningfully used in large cohort studies. While several concepts exist regarding the validation of biomarkers, there are no universally accepted validation criteria for dietary intake biomarkers. Given the above problems, the use of other omics (i.e., genomics, proteomics, and metagenomics) techniques in dietary biomarker analysis may serve to complement the metabolomic information that is normally collected for dietary biomarker studies.

## 7. Conclusions

Today, knowledge regarding valid BFVI in children and adolescents is still very limited. Among the 28 included studies, only 1 was using untargeted metabolomic analysis in young people, so explorative studies are missing in this age group. In this review, we found that carotenoid concentration in skin is a valuable biomarker for total FV intake for both young and adult populations. Common BFVI in adults (e.g., carotenoids and vitamin C) showed inconsistent results in children and adolescents. Diet–metabolite associations more plausible in children than adults include urinary hippuric acid with polyphenolic compound intake from FV and the combined *N*-methylnicotinic acid and acetylornithine with intake of beans. Proline betaine and *scyllo*-inositol are plausible biomarkers for citrus fruit and coconut intake, respectively, in children and adolescents. However, further studies are needed to assess their time–response, dose–response, and other validation aspects. In conclusion, future research is needed to obtain valid BFVI in the vulnerable group of children and adolescents, including discovery and in-depth validation studies.

## Figures and Tables

**Table 1:** Overview of the selected studies on biomarkers of fruit and vegetables intake in children and adolescents.

Dietary Factor	Sample Size	Country	Age (Years)	Study Design	Potential Biomarkers ^1^	Primary References
**Skin biomarkers**						
F/V	45 (20 boys)	USA	5–17	Cross-sectional	Total carotenoids	[24]
F/V	381 (193 boys)	USA	3–5	Cross-sectional	Total carotenoids	[25]
F/V	177 (83 boys)	USA	2–12	Cross-sectional	Total carotenoids	[26]
F/V	166 (62 boys)	USA	9–12	Cross-sectional	Total carotenoids	[27]
F/V	374 (N)	NM	NM	Noncontrolled dietary intervention	Total carotenoids	[28]
F/V	143 (68 boys)	USA	9–11	Cross-sectional	Total carotenoids	[29]
**Blood biomarkers**						
F/V	1192 (651 boys)	France, Greece, Lithuania, Norway, Spain, UK	6–11	Cross-sectional	Acetylornithine	[23]
F/V	45 (20 boys)	USA	5–17	Cross-sectional	Total carotenoids	[24]
F/V	166 (62 boys)	USA	9–12	Cross-sectional	Total carotenoids	[27]
F/V	81 (34 boys)	Danish	8–11	Cross-sectional	α- and β-Carotene, β-cryptoxanthin	[30]
F/V	97 (43 boys)	USA	6–10	Cross-sectional	Total carotenoids and vitamin C	[31]
F/V	122 boys	Spain	15–17	Cross-sectional	Total carotenoids	[32]
F/V	285 (boys 153)	USA	12–17	Cross-sectional	α-Carotene	[33]
F/V	93 (N)	Australia	5–12	Cross-sectional	β-Carotene, lycopene α-Carotene, cryptoxanthin	[34]
DGOV/green vegetable	210 (99 boys)	Brazil	9–13	Cross-sectional	β-carotene, 5-MTHF	[35]
Fruit/green-yellow vegetable	398 (214 boys)	Japan	10–11 and 13–14	Cross-sectional	β-Carotene, cryptoxanthin,	[36]
F/V	80 (23 boys)	Brazil	13.0 ± 1.1	Cross-sectional	β-Carotene	[37]
Fruit/root vegetable	207 (129 boys)	Finnish	1–3	Cohort study	α- and β-Carotene	[38]
Papaya	159 (81 boys)	Costa Rican	12–20	Cross-sectional	β-Cryptoxanthin	[39]
Momordica cochinchinensis (gac)	185 (N)	Vietnam	2–6	Controlled dietary intervention	α- and β-Carotene, retinol, lycopene, zeaxanthin	[40]
Amaranth	35 (N)	India	2–6	Controlled dietary intervention	Vitamin A	[41]
Sun-dried cowpea and amaranth leaves	152 (N)	Kenya	2.5–6	Controlled dietary intervention	β-Carotene, retinol	[42]
F/V	390 (163 boys)	Austria, Belgium, France, Germany, Greece, Hungary, Italy, Spain, and Sweden	12.5–17.5	Cross-sectional	Vitamin C, β-carotene	[43]
F/V	174 (82 boys)	Australia	0–17	Noncontrolled dietary intervention	β-Cryptoxanthin, lutein–zeaxanthin, vitamin C	[44]
F/V	45 (21 boys)	Brazil	6–10	Cross-sectional	Combination of β-carotene, retinol, vitamin C and α-tocopherol	[45]
Orange fruit/ dark-green leafy vegetable	238 (104 boys)	Indonesia	7–11	Controlled dietary intervention	Retinol, β-carotene, β-cryptoxanthin, lutein, lycopene	[46]
**Urinary biomarkers**						
F/V	330 (215 boys)	Australia	8	Cross-sectional	Potassium	[47]
Wild blueberry	15 (7 boys)	UK	7–10	Controlled dietary intervention	Hippuric acid, dihydro caffeic acid 3-*O*-sulfate	[48]
F/V	240 (120 boys)	Germany	9–10 and 12–15	Cross-sectional	Hippuric acid	[49]
FlavFV	287 (48% boys)	Germany	9–16	Cross-sectional	Hippuric acid	[50]
F/V	1192 (651 boys)	France, Greece, Lithuania, Norway, Spain, UK	6–11	Cross-sectional	Hippurate, proline betaine, NMNA, scyllo-inositol, acetate	[23]

^1^ These compounds were found to be significantly associated with fruit and vegetables intake; F/V: fruit and vegetables; N: sex not specified; NM: not mention; DGOV: dark green and orange vegetables; FlavFV: flavonoid intake from fruit and vegetables; 5-MTHF: 5-methyltetrahydrofolate; WWB: wild blueberry. NMNA: *N*-methylnicotinic acid.

**Table 2:** Summary of candidate BFVI for specific FV intake and reason for inclusion or exclusion.

Metabolites	HMDB ID	PubChem CID	Biofluid	Specificity	Reason	References
α-Tocopherol	1893	57393415	Blood	no	Common for many sources	[51]
5-MTHF	1396	135398561	Blood	no	Common for many sources	[52]
Potassium	586	5462222	Urine	no	Common for many sources	[53,54,55]
Acetate	-	175	Urine	no	Common for many sources	[56]
Hippuric acid	714	464	Urine	yes	Specific to polyphenolic compounds	[57,58]
Dihydro caffeic acid 3-*O*-sulfate	41721	49844181	Urine	yes	Combined biomarker	[59]
Proline betaine	4827	115244	Urine	yes	Specific to citrus	[60]
NMNA	-	5571	Urine	yes	Specific to beans	[61]
Acetylornithine	3357	6992102	Blood	yes	Combined biomarker	[62]
Scyllo-Inositol	6088	892	Urine	yes	Specific to coconut	[63]

5-MTHF: 5-methyltetrahydrofolate; NMNA: N-methylnicotinic acid. Criteria: (1) the marker has high specificity for the targeted food or food group, such as arsenobetaine for fish; (2) the compound is highly characteristic of the food investigated (e.g., markers that are very high in the targeted food compared with others, such as chlorogenic acid for coffee); and (3) the marker is not fully specific but could be used in a multimarker approach (e.g., tartaric acid is present in grapes but combined with ethyl glucuronide may provide a good estimation of wine intake.

**Table 3:** Overview of the validation criteria for candidate intake biomarkers.

	Substrate	Biomarker	Plausibility	Dose–Response	Time–Response	Robustness	Reliability	Stability	Analytical Performance	Reproducibility
Polyphenolic compounds	Urine	Dihydro caffeic acid 3-O-sulfate	Y	U	U	Y	U	U	U	U
Polyphenolic compounds	Urine	Hippuric acid	Y	U	U	U	Y	Y	Y	Y
Citrus	Urine	Proline betaine	Y	U	U	U	U	U	Y	U
Beans	Urine	NMNA	Y	U	U	U	U	U	Y	U
Coconut	Urine	Scyllo-inositol	Y	U	U	U	U	U	Y	U
Beans	Serum	Acetylornithine	Y	U	U	U	U	U	Y	U

NMNA: N-methylnicotinic acid. Q1: Is the marker compound plausible as a specific biomarker for the food or food group (chemical/biological plausibility)? Q2: Is there a dose–response relationship at relevant intake levels of the targeted food (quantitative aspect)? Q3: Is the biomarker kinetics described adequately to make a wise choice of sample type, frequency, and time window (time–response)? Q4: Has the marker been shown to be robust after intake of complex meals reflecting dietary habits of the targeted population (robustness)? Q5: Has the marker been shown to compare well with other markers or questionnaire data for the same food/food group (reliability)? Q6: Is the marker chemically and biologically stable during biospecimen collection and storage, making measurements reliable and feasible (stability)? Q7: Are analytical variability (CV%), accuracy, sensitivity, and specificity known to be adequate for at least one reported analytical method (analytical performance)? Q8: Has the analysis been successfully reproduced in another laboratory (reproducibility)? Y = yes; U = unknown.

## Supplementary Materials

The following are available online at https://www.mdpi.com/article/10.3390/metabo12020126/s1: Supplementary File S1: Search setup in three databases, Supplementary File S2: Biocross assessment tool, Supplementary File S3: Quality assessment of studies.

## References

[1] Lock K., Pomerleau J., Causer L., Altmann D.R., McKee M. (2005). The global burden of disease attributable to low consumption of fruit and vegetables: Implications for the global strategy on diet. Bull. World Health Organ..

[2] Jääskeläinen P., Magnussen C., Pahkala K., Mikkilä V., Kähönen M., Sabin M., Fogelholm M., Hutri-Kähönen N., Taittonen L., Telama R. (2012). Childhood Nutrition in Predicting Metabolic Syndrome in Adults: The Cardiovascular Risk in Young Finns Study. Diabetes Care.

[3] Tarasuk V.S., Brooker A.-S. (1997). Interpreting epidemiologic studies of diet-disease relationships. J. Nutr..

[4] Livingstone M.B., Robson P.J., Wallace J.M. (2004). Issues in dietary intake assessment of children and adolescents. Br. J. Nutr..

[5] Smith K.E. (2002). Who’s Minding the Kids?: Child Care Arrangements, Spring 1997.

[6] Iannotti R.J., Zuckerman A.E., Blyer E.M., O’Brien R.W., Finn J., Spillman D.M. (1994). Comparison of Dietary Intake Methods with Young Children. Psychol. Rep..

[7] Livingstone M.B.E., Robson P.J. (2000). Measurement of dietary intake in children. Proc. Nutr. Soc..

[8] Martin C.K., Newton R.L., Anton S.D., Allen H.R., Alfonso A., Han H., Stewart T., Sothern M., Willamson D. (2007). Measurement of children’s food intake with digital photography and the effects of second servings upon food intake. Eat. Behav..

[9] Baldrick F.R., Woodside J.V., Elborn J.S., Young I.S., McKinley M.C. (2011). Biomarkers of Fruit and Vegetable Intake in Human Intervention Studies: A Systematic Review. Crit. Rev. Food Sci. Nutr..

[10] Couillard C., Lemieux S., Vohl M.-C., Couture P., Lamarche B. (2016). Carotenoids as biomarkers of fruit and vegetable intake in men and women. Br. J. Nutr..

[11] Margalef M., Carres L.I., Pons Z., Bravo F.I., Muguerza B., Arola-Arnal A. (2016). Age related differences in the plasma kinetics of flavanols in rats. J. Nutr. Biochem..

[12] Agans R., Rigsbee L., Kenche H., Michail S., Khamis H.J., Paliy O. (2011). Distal gut microbiota of adolescent children is different from that of adults. FEMS Microbiol. Ecol..

[13] Ringel-Kulka T., Cheng J., Ringel Y., Salojärvi J., Carroll I., Palva A., De Vos W.M., Satokari R. (2013). Intestinal Microbiota in Healthy U.S. Young Children and Adults—A High Throughput Microarray Analysis. PLoS ONE.

[14] Mariat D., Firmesse O., Levenez F., Guimaraes V.D., Sokol H., Dore J., Corthier G., Furet J.-P. (2009). The Firmicutes/Bacteroidetes ratio of the human microbiota changes with age. BMC Microbiol..

[15] Radjabzadeh D., Boer C.G., Beth S.A., Van Der Wal P., Jong J.C.K.-D., Jansen M.A.E., Konstantinov S.R., Peppelenbosch M.P., Hays J., Jaddoe V.W.V. (2020). Diversity, compositional and functional differences between gut microbiota of children and adults. Sci. Rep..

[16] Beydoun M.A., Wang Y. (2009). Parent–child dietary intake resemblance in the United States: Evidence from a large representative survey. Soc. Sci. Med..

[17] Wang Y., Beydoun M.A., Li J., Liu Y., Moreno L.A. (2011). Do children and their parents eat a similar diet? Resemblance in child and parental dietary intake: Systematic review and meta-analysis. J. Epidemiol. Community Health.

[18] Maiani G., Castón M.J.P., Catasta G., Toti E., Cambrodón I.G., Bysted A., Granado-Lorencio F., Olmedilla-Alonso B., Knuthsen P., Valoti M. (2008). Carotenoids: Actual knowledge on food sources, intakes, stability and bioavailability and their protective role in humans. Mol. Nutr. Food Res..

[19] Dragsted L.O., Gao Q., Scalbert A., Vergères G., Kolehmainen M., Manach C., Brennan L., Afman L.A., Wishart D.S., Lacueva C.A. (2018). Validation of biomarkers of food intake—critical assessment of candidate biomarkers. Genes Nutr..

[20] Praticò G., Gao Q., Scalbert A., Vergères G., Kolehmainen M., Manach C., Brennan L., Pedapati S.H., Afman L.A., Wishart D.S. (2018). Guidelines for Biomarker of Food Intake Reviews (BFIRev): How to conduct an extensive literature search for biomarker of food intake discovery. Genes Nutr..

[21] Moher D., Liberati A., Tetzlaff J., Altman D.G., The PRISMA Group (2009). Preferred reporting items for systematic reviews and meta-analyses: The PRISMA Statement. PLoS Med..

[22] Wirsching J., Graßmann S., Eichelmann F., Harms L.M., Schenk M., Barth E., Berndzen A., Olalekan M., Sarmini L., Zuberer H. (2018). Development and reliability assessment of a new quality appraisal tool for cross-sectional studies using biomarker data (BIOCROSS). BMC Med. Res. Methodol..

[23] Lau C.-H.E., Siskos A., Maitre L., Robinson O., Athersuch T.J., Want E.J., Urquiza J., Casas M., Vafeiadi M., Roumeliotaki T. (2018). Determinants of the urinary and serum metabolome in children from six European populations. BMC Med..

[24] Aguilar S.S., Wengreen H.J., Lefevre M., Madden G.J., Gast J. (2014). Skin Carotenoids: A Biomarker of Fruit and Vegetable Intake in Children. J. Acad. Nutr. Diet..

[25] Scarmo S., Henebery K., Peracchio H., Cartmel B., Lin H., Ermakov I.V., Gellermann W., Bernstein P.S., Duffy V.B., Mayne S.T. (2012). Skin carotenoid status measured by resonance Raman spectroscopy as a biomarker of fruit and vegetable intake in preschool children. Eur. J. Clin. Nutr..

[26] Seguin-Fowler R.A., Hanson K.L., Marshall G.A., Belarmino E.H., Jilcott Pitts S.B., Kolodinsky J., Sitaker M., Ammerman A. (2021). Fruit and Vegetable Intake Assessed by Repeat 24 h Recalls, but Not by A Dietary Screener, Is Associated with Skin Carotenoid Measurements in Children. Nutrients.

[27] Nguyen L.M., Scherr R.E., Linnell J.D., Ermakov I.V., Gellermann W., Jahns L., Keen C.L., Miyamoto S., Steinberg F.M., Young H.M. (2015). Evaluating the relationship between plasma and skin carotenoids and reported dietary intake in elementary school children to assess fruit and vegetable intake. Arch. Biochem. Biophys..

[28] Whiteside-Mansell L., Swindle T., Davenport K. (2019). Evaluation of “Together, We Inspire Smart Eating” (WISE) Nutrition Intervention for Young Children: Assessment of Fruit and Vegetable Consumption with Parent Reports and Measurements of Skin Carotenoids as Biomarkers. J. Hunger Environ. Nutr..

[29] Martinelli S., Acciai F., Tasevska N., Ohri-Vachaspati P. (2021). Using the Veggie Meter in Elementary Schools to Objectively Measure Fruit and Vegetable Intake: A Pilot Study. Methods Protoc..

[30] Biltoft-Jensen A.P., Bysted A., Trolle E., Christensen T., Knuthsen P., Damsgaard C.T., Andersen L.F., Brockhoff P.B., Tetens I. (2012). Evaluation of Web-based Dietary Assessment Software for Children: Comparing reported fruit, juice and vegetable intakes with plasma carotenoid concentration and school lunch observations. Br. J. Nutr..

[31] Byers T., Treiber F., Gunter E., Coates R., Sowell A., Leonard S., Mokdad A., Jewell S., Miller D., Serdula M. (1993). The accuracy of parental reports of their children’s intake of fruits and vegetables: Validation of a food frequency questionnaire with serum levels of carotenoids and vitamins C, A, and E. Epidemiology.

[32] Notario-Barandiaran L., Navarrete-Muñoz E.-M., Valera-Gran D., Hernández-Álvarez E., Donoso-Navarro E., González-Palacios S., García-De-La-Hera M., Fernández M., Freire C., Vioque J. (2021). Biochemical Validation of a Self-Administered Food Frequency Questionnaire to Assess Diet Using Carotenoids and Vitamins E and D in Male Adolescents in Spain. Antioxidants.

[33] Neuhouser M.L., Rock C.L., Eldridge A.L., Kristal A.R., Patterson R.E., Cooper D.A., Neumark-Sztainer D., Cheskin L.J., Thornquist M.D. (2001). Serum concentrations of retinol, α-tocopherol and the carotenoids are influenced by diet, race and obesity in a sample of healthy adolescents. J. Nutr..

[34] Burrows T.L., Warren J.M., Colyvas K., Garg M.L., Collins C.E. (2008). Validation of Overweight Children’s Fruit and Vegetable Intake Using Plasma Carotenoids. Obesity.

[35] Hillesheim E., Toffano R.B.D., de Barros T.T., Salomão R.G., Mathias M.G., Coelho-Landell C.D.A., Almada M.O.R.D.V., Camarneiro J.M., Camelo-Junior J.S., Ued F.D.V. (2020). Biomarker-based validity of a food frequency questionnaire estimating intake in Brazilian children and adolescents. Int. J. Food Sci. Nutr..

[36] Okuda M., Sasaki S., Bando N., Hashimoto M., Kunitsugu I., Sugiyama S., Terao J., Hobara T. (2009). Carotenoid, Tocopherol, and Fatty Acid Biomarkers and Dietary Intake Estimated by Using a Brief Self-Administered Diet History Questionnaire for Older Japanese Children and Adolescents. J. Nutr. Sci. Vitaminol..

[37] Slater B., Enes C.C., López R.V.M., Damasceno N.R.T., Voci S.M. (2010). Validation of a food frequency questionnaire to assess the consumption of carotenoids, fruits and vegetables among adolescents: The method of triads. Cad. Saúde Pública.

[38] Prasad M., Takkinen H.-M., Uusitalo L., Tapanainen H., Ovaskainen M.-L., Alfthan G., Erlund I., Ahonen S., Åkerlund M., Toppari J. (2018). Carotenoid Intake and Serum Concentration in Young Finnish Children and Their Relation with Fruit and Vegetable Consumption. Nutrients.

[39] Irwig M., El-Sohemy A., Baylin A., Rifai N., Campos H. (2002). Frequent Intake of Tropical Fruits That Are Rich in β-Cryptoxanthin Is Associated with Higher Plasma β-Cryptoxanthin Concentrations in Costa Rican Adolescents. J. Nutr..

[40] Vuong L.T., Dueker S.R., Murphy S.P. (2002). Plasma β-carotene and retinol concentrations of children increase after a 30-d supplementation with the fruit Momordica cochinchinensis (gac). Am. J. Clin. Nutr..

[41] Lala V.R., Reddy V. (1970). Absorption of β-Carotene from Green Leafy Vegetables in Undernourished Children. Am. J. Clin. Nutr..

[42] Nawiri M.P., Nyambaka H., Murungi J.I. (2012). Sun-dried cowpeas and amaranth leaves recipe improves beta-carotene and retinol levels in serum and hemoglobin concentration among preschool children. Eur. J. Nutr..

[43] Vandevijvere S., Geelen A., Gonzalez-Gross M., Van’t Veer P., Dallongeville J., Mouratidou T., Dekkers A., Börnhorst C., Breidenassel C., Crispim S.P. (2012). Evaluation of food and nutrient intake assessment using concentration biomarkers in European adolescents from the Healthy Lifestyle in Europe by Nutrition in Adolescence study. Br. J. Nutr..

[44] Black A.P., Vally H., Morris P., Daniel M., Esterman A., Karschimkus C.S., O’Dea K. (2013). Nutritional impacts of a fruit and vegetable subsidy programme for disadvantaged Australian Aboriginal children. Br. J. Nutr..

[45] Collese T.S., De Moraes A.C.F., Rendo-Urteaga T., Luzia L.A., Rondó P.H.D.C., Marchioni D.M.L., Carvalho H.B. (2019). The Validity of Children’s Fruit and Vegetable Intake Using Plasma Vitamins A, C, and E: The SAYCARE Study. Nutrients.

[46] De Pee S., West C.E., Permaesih D., Martuti S., Muhilal, Hautvast J.G. (1998). Orange fruit is more effective than are dark-green, leafy vegetables in increasing serum concentrations of retinol and beta-carotene in schoolchildren in Indonesia. Am. J. Clin. Nutr..

[47] Jones G., Riley M.D., Whiting S. (2001). Association between urinary potassium, urinary sodium, current diet, and bone density in prepubertal children. Am. J. Clin. Nutr..

[48] Barfoot K.L., Istas G., Feliciano R.P., Lamport D.J., Riddell P., Rodriguez-Mateos A., Williams C.M. (2021). Effects of daily consumption of wild blueberry on cognition and urinary metabolites in school-aged children: A pilot study. Eur. J. Nutr..

[49] Krupp D., Doberstein N., Shi L., Remer T. (2012). Hippuric Acid in 24-Hour Urine Collections Is a Potential Biomarker for Fruit and Vegetable Consumption in Healthy Children and Adolescents. J. Nutr..

[50] Penczynski K.J., Krupp D., Bring A., Bolzenius K., Remer T., Buyken A.E. (2015). Relative validation of 24-hour urinary hippuric acid excretion as a biomarker for dietary flavonoid intake from fruit and vegetables in healthy adolescents. Eur. J. Nutr..

[51] Traber M.G. (2014). Vitamin E Inadequacy in Humans: Causes and Consequences. Adv. Nutr. Int. Rev. J..

[52] Iglesia I., Mouratidou T., Gonzalez-Gross M., Huybrechts I., Breidenassel C., Santabárbara J., Díaz L.-E., Hällström L., De Henauw S., Gottrand F. (2015). Foods contributing to vitamin B(6), folate, and vitamin B(12) intakes and biomarkers status in European adolescents: The HELENA study. Eur. J. Nutr..

[53] Huang Y., Van Horn L., Tinker L.F., Neuhouser M.L., Carbone L., Mossavar-Rahmani Y., Thomas F., Prentice R.L. (2014). Measurement error corrected sodium and potassium intake estimation using 24-h urinary excretion. Hypertension.

[54] Freedman L.S., Commins J.M., Moler J.E., Willett W., Tinker L.F., Subar A.F., Spiegelman D., Rhodes D., Potischman N., Neuhouser M.L. (2015). Pooled Results From 5 Validation Studies of Dietary Self-Report Instruments Using Recovery Biomarkers for Potassium and Sodium Intake. Am. J. Epidemiol..

[55] Park Y., Dodd K.W., Kipnis V., Thompson F.E., Potischman N., Schoeller D.A., Baer D.J., Midthune D., Troiano R., Bowles H. (2018). Comparison of self-reported dietary intakes from the Automated Self-Administered 24-h recall, 4-d food records, and food-frequency questionnaires against recovery biomarkers. Am. J. Clin. Nutr..

[56] Rawat S. (2015). Food Spoilage: Microorganisms and their prevention. Asian J. Plant Sci. Res..

[57] Lees H.J., Swann J.R., Wilson I.D., Nicholson J.K., Holmes E. (2013). Hippurate: The Natural History of a Mammalian–Microbial Cometabolite. J. Proteome Res..

[58] Pero R.W. (2010). Health Consequences of Catabolic Synthesis of Hippuric Acid in Humans. Curr. Clin. Pharmacol..

[59] Pereira-Caro G., Kay C.D., Clifford M.N., Crozier A. (2020). Flavanones. Dietary Polyphenols: Their Metabolism and Health Effects.

[60] Heinzmann S.S., Brown I.J., Chan Q., Bictash M., Dumas M.-E., Kochhar S., Stamler J., Holmes E., Elliott P., Nicholson J.K. (2010). Metabolic profiling strategy for discovery of nutritional biomarkers: Proline betaine as a marker of citrus consumption. Am. J. Clin. Nutr..

[61] Posma J.M., Garcia-Perez I., Heaton J.C., Burdisso P., Mathers J.C., Draper J., Lewis M., Lindon J.C., Frost G., Holmes E. (2017). Integrated Analytical and Statistical Two-Dimensional Spectroscopy Strategy for Metabolite Identification: Application to Dietary Biomarkers. Anal. Chem..

[62] Armstrong M.D. (1979). Nδ-acetylornithine and S-methylcysteine in blood plasma. Biochim. Biophys. Acta (BBA) Gen. Subj..

[63] Groenen P.M., Merkus H.M., Sweep F.C., Wevers R.A., Janssen F.S., Steegers-Theunissen R.P. (2003). Kinetics of myo-inositol loading in women of reproductive age. Ann. Clin. Biochem. Int. J. Lab. Med..

[64] Radtke M.D., Pitts S.J., Jahns L., Firnhaber G.C., Loofbourrow B.M., Zeng A., Scherr R.E. (2020). Criterion-Related Validity of Spectroscopy-Based Skin Carotenoid Measurements as a Proxy for Fruit and Vegetable Intake: A Systematic Review. Adv. Nutr..

[65] Parker R.S. (1996). Absorption, metabolism, and transport of carotenoids. FASEB J..

[66] Parker R.S., Swanson J.E., You C.-S., Edwards A.J., Huang T. (1999). Bioavailability of carotenoids in human subjects. Proc. Nutr. Soc..

[67] Furr H.C., Clark R.M. (1997). Intestinal absorption and tissue distribution of carotenoids. J. Nutr. Biochem..

[68] Umbreen H., Zia-Ul-Haq M. (2021). Carotenoids and Skin Diseases. Carotenoids: Structure and Function in the Human Body.

[69] Darvin M.E., Fluhr J.W., Caspers P., Van Der Pool A., Richter H., Patzelt A., Sterry W., Lademann J. (2009). In vivo distribution of carotenoids in different anatomical locations of human skin: Comparative assessment with two different Raman spectroscopy methods. Exp. Dermatol..

[70] Lademann J., Meinke M.C., Sterry W., Darvin M.E. (2011). Carotenoids in human skin. Exp. Dermatol..

[71] Zerres S., Stahl W. (2020). Carotenoids in human skin. Biochim. Biophys. Acta (BBA) Mol. Cell Biol. Lipids.

[72] Pennant M., Steur M., Moore C., Butterworth A., Johnson L. (2015). Comparative validity of vitamin C and carotenoids as indicators of fruit and vegetable intake: A systematic review and meta-analysis of randomised controlled trials. Br. J. Nutr..

[73] Tasevska N., Runswick S.A., Bingham S.A. (2006). Urinary Potassium Is as Reliable as Urinary Nitrogen for Use as a Recovery Biomarker in Dietary Studies of Free Living Individuals. J. Nutr..

[74] Olmedilla-Alonso B., Rodríguez-Rodríguez E., Beltrán-de-Miguel B., Estévez-Santiago R. (2020). Dietary beta-Cryptoxanthin and alpha-Carotene Have Greater Apparent Bioavailability Than beta-Carotene in Subjects from Countries with Different Dietary Patterns. Nutrients.

[75] Vázquez-Manjarrez N., Ulaszewska M., Garcia-Aloy M., Mattivi F., Praticò G., Dragsted L.O., Manach C. (2020). Biomarkers of intake for tropical fruits. Genes Nutr..

[76] EU (2009). Commission Directive 2008/128/EC of 22 December 2008 laying down specific purity criteria concerning colours for use in foodstuffs (codified version). Off. J. Eur. Union.

[77] Authority E.F.S. (2008). Use of Lycopene as a food colour-Scientific Opinion of the Panel on Food additives, Flavourings, Processing Aids and Materials in Contact with Food. EFSA J..

[78] Authority E.F.S. (2010). Revised exposure assessment for lycopene as a food colour. EFSA J..

[79] Clarke E.D., Rollo M.E., Collins C.E., Wood L., Callister R., Philo M., Kroon P.A., Haslam R.L. (2020). The Relationship between Dietary Polyphenol Intakes and Urinary Polyphenol Concentrations in Adults Prescribed a High Vegetable and Fruit Diet. Nutrients.

[80] Ogata M., Taguchi T. (1988). Simultaneous determination of urinary creatinine and metabolites of toluene, xylene, styrene, ethylbenzene and phenol by automated high performance liquid chromatography. Int. Arch. Occup. Environ. Health.

[81] Tomokuni K., Ogata M. (1972). Direct Colorimetric Determination of Hippuric Acid in Urine. Clin. Chem..

[82] Pujos-Guillot E., Hubert J., Martin J.-F., Lyan B., Quintana M., Claude S., Chabanas B., Rothwell J., Bennetau-Pelissero C., Scalbert A. (2013). Mass Spectrometry-based Metabolomics for the Discovery of Biomarkers of Fruit and Vegetable Intake: Citrus Fruit as a Case Study. J. Proteome Res..

[83] Lang R., Lang T., Bader M., Beusch A., Schlagbauer V., Hofmann T. (2017). High-Throughput Quantitation of Proline Betaine in Foods and Suitability as a Valid Biomarker for Citrus Consumption. J. Agric. Food Chem..

[84] Gibbons H., Michielsen C., Rundle M., Frost G., McNulty B.A., Nugent A.P., Walton J., Flynn A., Gibney M.J., Brennan L. (2017). Demonstration of the utility of biomarkers for dietary intake assessment; proline betaine as an example. Mol. Nutr. Food Res..

[85] Ashihara H., Ludwig I.A., Katahira R., Yokota T., Fujimura T., Crozier A. (2014). Trigonelline and related nicotinic acid metabolites: Occurrence, biosynthesis, taxonomic considerations, and their roles in planta and in human health. Phytochem. Rev..

[86] Evans L.S., Tramontano W.A. (1984). Trigonelline and promotion of cell arrest in G2 of various legumes. Phytochemistry.

[87] Perera T., Young M.R., Zhang Z., Murphy G., Colburn N.H., Lanza E., Hartman T.J., Cross A.J., Bobe G. (2015). Identification and monitoring of metabolite markers of dry bean consumption in parallel human and mouse studies. Mol. Nutr. Food Res..

[88] Kinnard R., Narasimhan B., Pliskamatyshak G., Murthy P. (1995). Characterization of Scyllo-Inositol-Containing Phosphatidylinositol in Plant Cells. Biochem. Biophys. Res. Commun..

[89] Ichimura K., Kohata K., Yamaguchi Y., Douzono M., Ikeda H., Koketsu M. (2000). Identification ofL-Inositol and Scyllitol and Their Distribution in Various Organs in Chrysanthemum. Biosci. Biotechnol. Biochem..

[90] Majeed M., Badmaev V. (2010). Coconut Water and Its the Method of Preparation. https://www.researchgate.net/publication/260064797_COCONUT_WATER_AND_ITS_THE_METHOD_OF_PREPARATION.

[91] Zha H., Cai Y., Yin Y., Wang Z., Li K., Zhu Z.-J. (2018). SWATHtoMRM: Development of High-Coverage Targeted Metabolomics Method Using SWATH Technology for Biomarker Discovery. Anal. Chem..

[92] Johnson C.H., Ivanisevic J., Siuzdak G. (2016). Metabolomics: Beyond biomarkers and towards mechanisms. Nat. Rev. Mol. Cell Biol..

