# Supplementary material for: Assessment of Fruit and Vegetables Intake with Biomarkers in Children and Adolescents and Their Level of Validation: A Systematic Review
Source: Metabolites. 2022 Jan 28;12(2):126. doi: 10.3390/metabo12020126 (PMC8876138; doi:10.3390/metabo12020126)
Supplement: Supplementary file 1 [file metabolites-12-00126-s001.zip › Supplementary File S1 search set-up in three databases.pdf]

Table S1: The full search strategy on PubMed, the database coverage was 1946 to present and was searched on 15 January 2021.

Search	Query	Result
17	#16 and #10 and #4	1662
16	#12 or #13 or #14 or #15	4,160,999
15	"Adolescent"[Mesh]	2,061,820
14	"Child"[Mesh]	1,939,644
13	"Infant"[Mesh]	1,154,174
12	child[Title/Abstract] OR youth[Title/Abstract] OR juvenile*[Title/Abstract] OR preschool[Title/Abstract] OR boy*[Title/Abstract] OR girl*[Title/Abstract] OR teenage*[Title/Abstract] OR children[Title/Abstract] OR childhood[Title/Abstract] OR adolescence[Title/Abstract] OR infant*[Title/Abstract] OR infancy[Title/Abstract] OR adolescent*[Title/Abstract]	2,106,366
10	#6 or #7 or #8 or #9	1,108,867
9	"Metabolome"[Mesh]	10,905
8	"Metabolomics"[Mesh]	17,891
7	"Biomarkers"[Mesh]	767,036
6	(urine[Title/Abstract] OR urinary[Title/Abstract] OR plasma[Title/Abstract] OR serum*[Title/Abstract] OR excretion*[Title/Abstract] OR blood[Title/Abstract] OR skin[Title/Abstract] OR saliva[Title/Abstract]) AND (biomarker*[Title/Abstract] OR marker*[Title/Abstract] OR metabolite*[Title/Abstract] OR biokinetics[Title/Abstract] OR biotransformation[Title/Abstract] OR bioavailability[Title/Abstract] OR ADME[Title/Abstract])	461,623
4	#1 or #2 or #3	439,554
3	"Vegetables"[Mesh]	31,705
2	"Fruit"[Mesh]	106,704
1	fruit*[Title/Abstract] OR vegetable*[Title/Abstract] OR citrus[Title/Abstract] OR berry[Title/Abstract] OR berries[Title/Abstract] OR apple*[Title/Abstract] OR grape*[Title/Abstract] OR pear*[Title/Abstract] OR orange*[Title/Abstract] OR banana*[Title/Abstract] OR mango*[Title/Abstract] OR pineapple*[Title/Abstract] OR tomato*[Title/Abstract] OR potato*[Title/Abstract] OR onion*[Title/Abstract] OR pea*[Title/Abstract] OR leek*[Title/Abstract] OR spinach*[Title/Abstract] OR lettuce*[Title/Abstract] OR endive*[Title/Abstract] OR asparagus*[Title/Abstract] OR kale[Title/Abstract] OR Brassica[Title/Abstract] OR cabbage*[Title/Abstract] OR "cruciferous vegetable*"[Title/Abstract]	366,806

Table S2: The full search strategy on Scopus, the database coverage was 1946 to present and was searched on 15 January 2021.

Search	Query	result
4	(TITLE-ABS (fruit* OR vegetable* OR citrus OR berry OR berries OR apple* OR grape* OR pear* OR orange* OR banana* OR mango* OR pineapple* OR tomato* OR potato* OR onion* OR pea* OR leek* OR spinach* OR lettuce* OR endive* OR asparagus* OR kale OR brassica OR cabbage* OR "cruciferous vegetable*")) AND (TITLE-ABS ((urine OR urinary OR plasma OR serum* OR excretion* OR blood OR skin OR saliva) AND (biomarker* OR marker* OR metabolite* OR biokinetics OR biotransformation OR bioavailability OR adme OR metabolom*))) AND (TITLE-ABS (child OR youth OR juvenile* OR preschool OR boy* OR girl* OR teenage* OR children OR childhood OR adolescence OR infant* OR infancy OR adolescent*)) AND NOT INDEX (medline)	394
3	(TITLE-ABS (fruit* OR vegetable* OR citrus OR berry OR berries OR apple* OR grape* OR pear* OR orange* OR banana* OR mango* OR pineapple* OR tomato* OR potato* OR onion* OR pea* OR leek* OR spinach* OR lettuce* OR endive* OR asparagus* OR kale OR brassica OR cabbage* OR "cruciferous vegetable*")) AND (TITLE-ABS ((urine OR urinary OR plasma OR serum* OR excretion* OR blood OR skin OR saliva) AND (biomarker* OR marker* OR metabolite* OR biokinetics OR biotransformation OR bioavailability OR adme OR metabolom*))) AND (TITLE-ABS (child OR youth OR juvenile* OR preschool OR boy* OR girl* OR teenage* OR children OR childhood OR adolescence OR infant* OR infancy OR adolescent*))	2009
2	TITLE-ABS ((urine OR urinary OR plasma OR serum* OR excretion* OR blood OR skin OR saliva) AND (biomarker* OR marker* OR metabolite* OR biokinetics OR biotransformation OR bioavailability OR adme OR metabolom*))	525,596
1	TITLE-ABS (fruit* OR vegetable* OR citrus OR berry OR berries OR apple* OR grape* OR pear* OR orange* OR banana* OR mango* OR pineapple* OR tomato* OR potato* OR onion* OR pea* OR leek* OR spinach* OR lettuce* OR endive* OR asparagus* OR kale OR brassica OR cabbage* OR "cruciferous vegetable*")	2,547,671

Table S3: The full search strategy on Web of Science, the database coverage was 1900 to present and was searched on 15 January 2021.

research	query	result
10	#9 AND #6 AND #3 *Indexes=SCI-EXPANDED, SSCI, A&HCI, CPCI-S, CPCI-SSH, BKCI-S, BKCI-SSH, ESCI, CCR-EXPANDED, IC Timespan=All years*	1518
9	#7 OR #8 *Indexes=SCI-EXPANDED, SSCI, A&HCI, CPCI-S, CPCI-SSH, BKCI-S, BKCI-SSH, ESCI, CCR-EXPANDED, IC Timespan=All years*	2,437,047
8	AB=(child OR youth OR juvenile* OR preschool OR boy* OR girl* OR teenage* OR children OR childhood OR adolescence OR infant* OR infancy OR adolescent*) *Inde xes=SCI-EXPANDED, SSCI, A&HCI, CPCI-S, CPCI-SSH, BKCI-S, BKCI-SSH, ESCI, CCR-EXPANDED, IC Timespan=All years*	1,615,500
7	TI=(child OR youth OR juvenile* OR preschool OR boy* OR girl* OR teenage* OR c hildren OR childhood OR adolescence OR infant* OR infancy OR adolescent*) *Inde xes=SCI-EXPANDED, SSCI, A&HCI, CPCI-S, CPCI-SSH, BKCI-S, BKCI-SSH, ESCI, CCR-EXPANDED, IC Timespan=All years*	1,558,422
6	#4 OR #5 *Indexes=SCI-EXPANDED, SSCI, A&HCI, CPCI-S, CPCI-SSH, BKCI-S, BKCI-SSH, ESCI, CCR-EXPANDED, IC Timespan=All years*	415,195
5	AB=((urine OR urinary OR plasma OR serum* OR excretion* OR blood OR skin OR saliva) and (biomarker* OR marker* OR metabolite* OR biokinetics OR biotransformation OR bioavailability OR ADME OR metabolom*)) *Indexes=SCI-EXPANDED, SSCI, A&HCI, CPCI-S, CPCI-SSH, BKCI-S, BKCI-SSH, ESCI, CCR-EXPANDED, IC Timespan=All years*	384,910
4	TI=((urine OR urinary OR plasma OR serum* OR excretion* OR blood OR skin OR saliva) and (biomarker* OR marker* OR metabolite* OR biokinetics OR biotransformation OR bioavailability OR ADME OR metabolom*)) *Indexes=SCI-EXPANDED, SSCI, A&HCI, CPCI-S, CPCI-SSH, BKCI-S, BKCI-SSH, ESCI, CCR-EXPANDED, IC Timespan=All years*	61,077
3	#1 OR #2 *Indexes=SCI-EXPANDED, SSCI, A&HCI, CPCI-S, CPCI-SSH, BKCI-S, BKCI-SSH, ESCI, CCR-EXPANDED, IC Timespan=All years*	2,010,997
2	TI=(fruit* OR vegetable* OR citrus OR berry OR berries OR apple* OR grape* OR p ear* OR orange* OR banana* OR mango* OR pineapple* OR tomato* OR potato* O R onion* OR pea* OR leek* OR spinach* OR lettuce* OR endive* OR asparagus* OR kale OR Brassica OR cabbage* OR "cruciferous vegetable*") *Indexes=SCI-EXPANDED, SSCI, A&HCI, CPCI-S, CPCI-SSH, BKCI-S, BKCI-SSH, ESCI, CCR-EXPANDED, IC Timespan=All years*	1,742,315
1	AB=(fruit* OR vegetable* OR citrus OR berry OR berries OR apple* OR grape* OR pear* OR orange* OR banana* OR mango* OR pineapple* OR tomato* OR potato* OR onion* OR pea* OR leek* OR spinach* OR lettuce* OR endive* OR asparagus* OR kale OR Brassica OR cabbage* OR "cruciferous vegetable*") *Indexes=SCI-EXPANDED, SSCI, A&HCI, CPCI-S, CPCI-SSH, BKCI-S, BKCI-SSH, ESCI, CCR-EXPANDED, IC Timespan=All years*	583,926
