# Supplementary material for: Assessment of Fruit and Vegetables Intake with Biomarkers in Children and Adolescents and Their Level of Validation: A Systematic Review
Source: Metabolites. 2022 Jan 28;12(2):126. doi: 10.3390/metabo12020126 (PMC8876138; doi:10.3390/metabo12020126)
Supplement: Supplementary file 1 [file metabolites-12-00126-s001.zip › Supplementary File S2 BIO-CROSS assessment tool.pdf]

N	Question	Study quality feature
1.	**<u>Was the research question in this paper clearly stated?</u>**<br>-Biomarker description<br>-Study objectives<br>-Pre-specified hypotheses	**Hypothesis/Objective (0,1,2,3)**
2.	**<u>Was the study design clearly specified and defined?</u>**<br>-Characteristics of the study participants<br>-Inclusion and exclusion criteria	**Study population selection (0,1,2)**
3.	**<u>Was the study population representative of the target/reference population under investigation?</u>**<br>-Sampling frame (study population source)<br>-Participation rate (i.e. eligible persons at least 50%)?<br>-Sample size justification/ power description	**Study population representativeness (0,1,2,3)**
4.	**<u>Was the study population clearly described?</u>**<br>-Study population characteristics (demographic, clinical and social)<br>-Exposures and potential confounders<br>-Were missing values and strategies to deal with missing data reported	**Study population characteristics (0,1,2,3)**
5.	**<u>Were statistical analyses adequately described?</u>**<br>-Statistical test for correlation reported (Spearman/Pearson)<br>-Univariable or multivariable linear regression<br>-Key potential confounding variables measured and adjusted statistically in reported analyses<br>-Significance level tested and reported	**Statistical analysis (0,1,2,3)**
6.	**<u>Are the results interpreted in the context of the pre-specified hypotheses?</u>**<br>-Interpretation of the results considering results from similar studies<br>-Biological context	**Data interpretation (0,1,2)**
7.	**<u>Was the biological material and assay methods clearly described?</u>**<br>- Assay methods (preservation and storage, detailed protocol, including specific reagents or kits used<br>-Quality control procedures	**Specimen characteristics and assay methods (0,1,2,3,4)**

	-Reproducibility assessments<br>-Quantitation methods and scoring and reporting protocols	
8.	**Were the laboratory measurements of the biomarkers clearly reported?**<br>-Laboratory/place of measurement<br>-Blinded analysis	**Laboratory measurement (0,1,2)**
9.	**Were biomarkers adequately handled in statistical analysis and in data presentation?**<br>--Biomarker variable distribution<br>-Outlier detection and handling<br>-Possible errors resulting from measurement inaccuracies discussed	**Biomarker data modeling (0,1,2,3)**

Every positive evaluation grade 1 point
Quality Rating: strong (18-25 points), moderate (9-17 Points), or Poor (≤ 8 Points)
